# Integrated mental health care and vocational rehabilitation intervention to improve return to work rates for people on sick leave due to common mental and functional disorders (IBBIS-II)—a study protocol for a randomized clinical trial

**DOI:** 10.1186/s13063-022-06718-7

**Published:** 2022-09-30

**Authors:** Thomas Nordahl Christensen, Chalotte Heinsvig Poulsen, Bea Kolbe Ebersbach, Lene Falgaard Eplov

**Affiliations:** Copenhagen Research Center for Mental Health (CORE), Mental Health Center Copenhagen, Copenhagen, Denmark

**Keywords:** Common mental illness, Integrated mental health care, Vocational rehabilitation, Randomized controlled trial

## Abstract

**Background:**

Mental illness has an estimated financial burden on the Danish economy of 3.4% of the gross national product every year due to lost productivity, social benefits, and healthcare costs, and approximately 50% of people receiving long-term sickness benefits have a common mental illness. Furthermore, a significant treatment gap exists where less than 30% are treated for their mental illness. The primary objective of the randomized trial is to examine whether people on sick leave with a diagnosis of anxiety, depression, stress, personality disorders, or functional disorders return to work faster and have higher job retention if they receive an integrated and optimized vocational rehabilitation and mental health care intervention, compared to people who receive the standard mental health care and vocational rehabilitation service.

**Methods:**

The trial is designed as an investigator-initiated, randomized, two-group parallel, assessor-blinded, superior trial. A total of 900 participants with a common mental illness will randomly be assigned into two groups: (1) IBBIS-II, consisting of integrated mental health care and vocational rehabilitation, or (2) service as usual (SAU), at two sites in Denmark. The primary outcome is the difference between the two groups in time to return to work (RTW) at 12 months using data from the Danish Register for Evaluation of Marginalization (DREAM) database.

**Discussion:**

This study will contribute with new knowledge on vocational recovery and integrated vocational and health care interventions in a Scandinavian context.

**Trial registration:**

ClinicalTrials.govNCT04432129. Registered on June 16, 2020

## Administrative information

Note: The numbers in curly brackets in this protocol refer to the SPIRIT checklist item numbers. The order of the items has been modified to group similar items.

**Table:** 

Title {1}	Integrated mental health care and vocational rehabilitation intervention to improve return to work rates for people on sick leave due to common mental and functional disorders (IBBIS-II)—a study protocol for a randomized clinical trial
Trial registration {2a and 2b}.	ClinicalTrials.gov, NCT04432129
Protocol version {3}	Version 3.1 27.5.2020
Funding {4}	The IBBIS-II trial is financed by the Danish Agency of Labour Market and Recruitment (STAR)
Author details {5a}	All authors are affiliated to Copenhagen Research Center for Mental Health (CORE), Mental Health Center Copenhagen, Denmark
Name and contact information for the trial sponsor {5b}	Research consultant, PhD, Lene Falgaard Eplov (Principal investigator) Copenhagen Research Center for Mental Health (CORE), Recovery & Inclusion, Mental Health Center Copenhagen Gentofte Hospitals Vej 15, Entrance 3A, 4^th^ floor 2900 Hellerup, DK Email: lene.falgaard.eplov@regionh.dkPhone: 0045 38647461
Role of sponsor {5c}	The trial sponsor developed the trial and participated in planning and designing the intervention and the study design. The IBBIS-II intervention is funded by STAR and is carried out at two sites in Denmark. The funder has no role in conducting the analysis, interpretation of the data, or decision to publish the study. DISCUS has the overall administrative responsibility for the implementation and the operational part of the project. Deloitte is responsible for data management, randomization, and data collection. At CORE, the evaluation is headed by the trial sponsor, who carries the responsibility for the evaluation of the data material. Metrica (Director and Professor Michael Rosholm and Michael Svarer) are advisors on the design of the evaluation and data analysis. Deloitte will have authority over data collection. CORE will have authority for the analysis and interpretation of the data, writing of the report and articles, and the decision to submit the report and articles for publication. DISCUS, Deloitte, and STAR will not take part in the decisions regarding data analysis nor the interpretation or publication of the results.

## Introduction

### Background and rationale {6a}

Mental illness has an estimated financial burden on the Danish economy of 3.4% of gross national product every year due to lost productivity, social benefits, and healthcare costs and approximately 50% of all people receiving long-term sickness benefits have a common mental disorder. Furthermore, a significant treatment gap exists where less than 30% are treated for their common mental illness [1]. On this basis, the Organization for Economic Co-operation and Development (OECD) has recommended that employment-oriented mental health care should be developed in Denmark, in addition to experimenting with ways to integrate health and employment services [1]. These recommendations, and the sickness benefit reform from 2015, led the Danish Agency for Labour Market and Recruitment (STAR) to launch the “Integrated Employment and Treatment Initiative for Sickness Beneficiaries (IBBIS)” project in 2016. The IBBIS intervention was designed to provide coherent and coordinated mental health care and vocational rehabilitation services to improve the return to work rates for people with stress, depression, and anxiety, and the intervention was evaluated in two randomized controlled trials (RCTs) [2, 3].

As a next step, the initiative was expanded to also provide service to people with personality disorders and functional disorders. The two new groups were selected based on practical knowledge from Jobcenter’s and the literature. A Danish survey showed that the vast majority of people on long-term sick leave suffered from depression or anxiety, but the two following groups were people with somatoform disorders (7%) and personality disorders (5%) [4]. Moreover, a study found that 14% of people with at least 8 weeks of sick leave had a functional disorder [5]. Although personality disorders only account for 5% of people on long-term sick leave, personality disorders develop from early adulthood and have major significance for the ability to work, in addition to a high risk of later onset of anxiety and depression [6].

To include the two new diagnostic groups, the manual-based IBBIS service was revised based on two systematic reviews, as well as drawing on practical experiences gathered from IBBIS-I [7]. Among other things, the vocational rehabilitation service and the organization of the intervention were updated, and the manual was expanded to also include work-related cognitive behavioral treatment (CBT) and treatment for people with personality disorders and functional disorders. The IBBIS-II services are now delivered by Copenhagen and Aarhus municipalities in a collaboration with Psychotherapeutic Clinic, Mental Health Services in the Capital Region of Denmark and Clinical Social Medicine & Rehabilitation, Central Jutland Region, respectively. Due to the extension of two new diagnostic groups and the changes in the delivered services, the effect of the intervention is evaluated in a new RCT which started the inclusion of participants in August 2019. However, due to COVID-19 and the lockdown in Denmark, it was not possible to deliver the IBBIS-II service as intended and complete the study as planned. Because the participants did not receive sufficient support from August 2019 to June 2020, this period was considered as a pilot phase. The present trial started the inclusion of participants in June 2020.

### Objectives {7}

The aim of the randomized trial is to examine whether people on sick leave with a diagnosis of anxiety, depression, stress, personality disorders, and functional disorders return to work faster and have higher job retention if they receive an integrated and optimized vocational rehabilitation and mental health care intervention, compared to people who receive service as usual (SAU), i.e., the standard mental health care and vocational rehabilitation service. The hypothesis is that the IBBIS-II intervention is superior to SAU in return to work (RTW) time at 12 months.

Furthermore, the aim is to include enough participants to analyze whether there are differences in RTW time within the diagnostic subgroup’s depression and stress. We will examine whether these specific subgroups have faster RTW rates and have higher job retention when offered the integrated IBBIS-II intervention compared with participants who receive standard mental health care and vocational rehabilitation.

### Trial design {8}

The trial is designed as an investigator-initiated, randomized, two-group parallel, assessor-blinded, superior trial. Participants in the pilot phase and the main trial will be randomized 1:1 into the two arms of the trial. Only participants in the main trial will be included in the primary analysis.

**Fig. 1:**
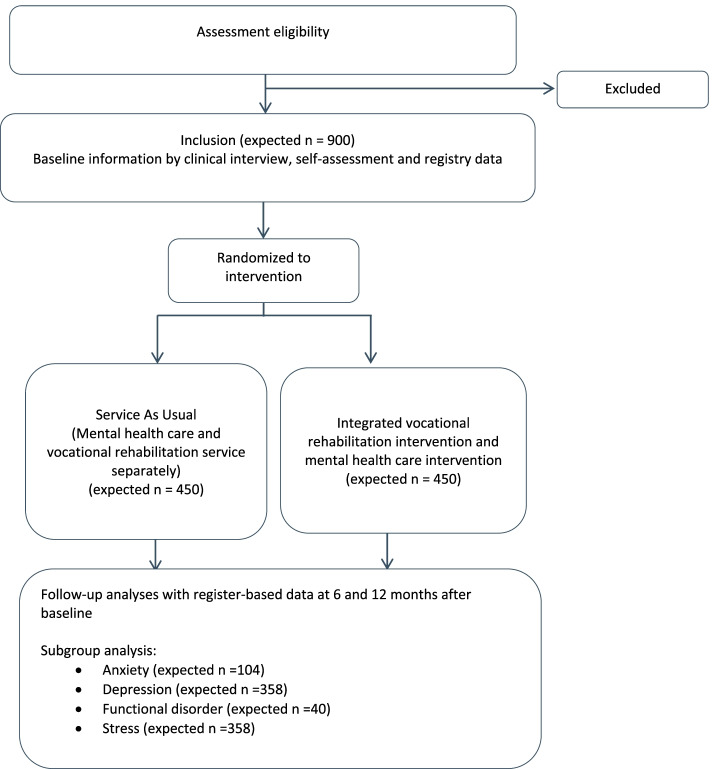
Flowchart of the RCT

Figure 1 depicts the flowchart of the trial and the number of participants expected to be included within the groups listed above with a ratio of 1:1 between participants in the two interventions and enough power to examine the effect of the diagnostic subgroup’s depression and stress. The follow-up is only planned through register-based data. The trial will be reported according to the modified CONSORT criteria for non-pharmacological trials [8]

## Methods: participants, interventions, and outcomes

### Study setting {9}

The interventions will be delivered by a cross-sector and multidisciplinary IBBIS-II team that is organized in collaboration between the mental health services and the vocational services (job centers) in two Danish regions, the Capital Region of Denmark in Copenhagen and the Central Region of Denmark in Aarhus. Participants are referred to the study by a sickness benefit caseworker from the job centers at the two study sites, and the interventions are provided at the job centers.

### Eligibility criteria {10}

All participants must be recipients of sickness benefits and on sick leave from a job or unemployed for a minimum of 4 weeks due to anxiety, depression, stress, personality disorders, or functional disorders.

The following are the inclusion criteria:

- Anxiety, depression, stress, personality disorder, or functional disorder diagnosed at a structured diagnostic interview based on the Mini-International Neuropsychiatric Interview conducted by the IBBIS team [9].
- Sickness benefit recipient at baseline; on sick leave from a job or unemployed for a minimum of 4 weeks
- Resident in Copenhagen or Aarhus municipalities
- Speak sufficient Danish to participate in interviews and complete questionnaires without an interpreter
- Aged ≥ 18 years
- Has given informed written consent

The following are the exclusion criteria:

- Pregnant
- High degree of suicidal ideation
- Dementia
- Abuse of alcohol or other drugs to the degree that participation in therapy is not possible
- A need for mental health treatment in secondary sector care
- An unstable somatic condition that is too severe for participation in the project
- Participation in any psychotherapy or psychotherapy-like treatment outside the IBBIS project, if the participant is randomized to receive the IBBIS intervention

### Who will take informed consent? {26a}

Participants are referred to the IBBIS-II team, by the case worker in the job center, if the general practitioner has stated a mental illness or functional disorder or suspicion thereof or if the participant or the caseworker suspects a mental or functional disorder and the participant has given informed consent to participate in the health assessment. Then, the sickness benefit caseworker at the job center acquires a health professional assessment at the regional IBBIS-II intervention. If the participant meets the criteria for a diagnosis of anxiety, depression, stress, personality disorder, or functional disorder in the health assessment, the participant can be included and randomized in the trial. Before randomization, the participant will be informed orally and in writing about the trial, and assigned informed consent will be attained at the job center. The individual information contains adequate information about the IBBIS-II trial, and the participants are informed that participation in the trial is voluntary, that the trial is not estimated to have any adverse effects, and that they can withdraw from the trial at any time, without consequence for future mental health or employment services.

### Additional consent provisions for collection and use of participant data and biological specimens {26b}

This trial does not involve collecting biological specimens for storage

### Interventions

#### Explanation for the choice of comparators {6b}

We aim to investigate if the IBBIS-II intervention is superior to SAU, i.e., standard mental health care and vocational rehabilitation service. Therefore, we chose to compare the IBBIS-II intervention to SAU alone.

#### Intervention description {11a}

The IBBIS-II service is thoroughly described in the manual and educational material. The service is briefly described below. The IBBIS-II service is delivered by two teams—one in Copenhagen and one in Aarhus. Each team has a team leader and consists of the following:

- Employment specialists who operate as caseworkers and are employed by job centers
- Care managers consisting of nurses, occupational therapists, physiotherapists, etc. with experience in mental health care and a minimum of 1 year of training in cognitive behavioral therapy
- Other healthcare professionals (psychologists, psychiatrists, specialists in social medicine, physiotherapists, and instructors in mindfulness-based stress reduction)

Healthcare professionals are employed by the two collaborating regions, and employment specialists are employed by the job centers in the two municipalities. Care managers have a maximum momentary caseload of 25, and employment specialists have a maximum momentary caseload of 30.

The entire staff has received training in the IBBIS-II service prior to the initiation of the study, and the employment specialists and care managers receive ongoing supervision.

The IBBIS-II services have the following core elements:

1. Assessment
  - All participants referred to the service receive a mental health assessment.
2. Planned integrated service
  - The participant has the same employment specialists and care manager throughout the intervention.
  - A joint plan is made with shared decisions between the participant, the employment specialist, and the care manager.
  - There is a focus on disclosure (openness about illness) and involvement of relatives and significant others.
3. Vocational rehabilitation
  - Focus on RTW with ongoing assessment of job goals, competencies, and need of support.
  - Participants receive help in contacting existing employers (employed) and potential employers (unemployed).
  - Unemployed participants are offered an individualized job search effort.
  - The focus is on competitive jobs within the community.
4. The treatment
  - Is based on stepped care and uses structured treatment guidelines. The stepped care includes the following:
    i. Psychoeducation
    ii. Work-related cognitive behavioral therapy for depression and anxiety disorders and special stress interventions for stress and stress-related disorders
    iii. Psychotherapy, physiotherapy, and medical treatment
5. Ongoing support
  - When the participant has obtained work the team continues to offer support.

#### Service as usual (SAU)

The control group receives SAU, i.e., the treatment and employment interventions that are usually offered to citizens who are on sick leave due to depression, anxiety, stress, personality disorders, and functional disorders in the regions and municipalities where IBBIS-II is being tested.

#### Criteria for discontinuing or modifying allocated interventions {11b}

There will be no specific criteria for discontinuing or modifying allocated interventions.

#### Strategies to improve adherence to interventions {11c}

The IBBIS-II team delivers the intervention IBBIS-II mental health care integrated with IBBIS-II vocational rehabilitation. The interventions are carried out with emphasis on participant involvement through shared decision-making to improve participant satisfaction [10] and involvement of the participants’ relatives. Continuously, adherence to intervention protocol is monitored. A fidelity scale is developed and used for biannual fidelity reviews to ensure program adherence and continuous focus on program implementation and improvement.

#### Relevant concomitant care permitted or prohibited during the trial {11d}

Participation in any psychotherapy or psychotherapy-like treatment outside the IBBIS-II project is unwanted during the trial if the participant is randomized to receive the IBBIS intervention.

#### Provisions for post-trial care {30}

There is no anticipated harm and compensation for trial participation

### Outcomes {12}

The effects of the intervention are measured on both primary and secondary outcomes. Measurements of primary, secondary, and explorative outcomes are performed at baseline and after 6 and 12 months using data from several Danish registers. The social service benefit data is obtained from the Danish Register for Evaluation of Marginalization (DREAM) database. The DREAM database is administered by STAR and can be linked to a range of different Danish national registers, including the Danish Income Register, the Danish Psychiatric Central Research Register, the National Central Patient Register, and the Health Insurance Register. The questionnaire, including symptom level, functioning, self-efficacy, and quality of life, is obtained from validated self-assessed questionnaires at baseline and not at 6 and 12 months. Baseline measurements are completed immediately after visitation at the job center. The primary, secondary, and exploratory outcomes are presented in Table 1.

**Table 1:** Primary, secondary, and exploratory outcomes

	Data source	Outcome	Baseline	6 months	12 months
Primary	DREAM data	Time to RTW (≤ 4 weeks of continuous work without receiving sickness benefit)	X		X
Secondary	DREAM data	Proportion of unsupported competitive employment	X		X
Exploratory	DREAM data	Time to RTW (≤ 4 weeks of continuous work without receiving sickness benefit)	X	X	
	DREAM data	Proportion of unsupported competitive employment	X	X	
	DREAM data	Proportion of supported competitive employment	X	X	
	DREAM data	The duration from RTW to potential new sick leave of more than 4 weeks	X		X
	DREAM data	Number of weeks of work from baseline to follow-up	X	X	X
	E-income data	Salary income from competitive employment from baseline to follow-up	X	X	X
	Psychiatric Central Research Register	Number of admissions, inpatient days, and outpatient contacts at the psychiatric hospitals	X	X	X
	The National Central Patient Register	Number of admissions, inpatient days, and outpatient contacts at the somatic hospitals	X	X	X
	Health Insurance Register	Number of contacts with private health care professionals under the health insurance	X	X	X
	Questionnaire	Difference in symptoms measured with the Four-Dimensional Symptom Questionnaire (4DSQ)	X		
	Questionnaire	Difference in social and work-related function measured with the Work and Social Adjustment Scale (WSAS)	X		
	Questionnaire	Burnout symptoms measured by Karolinska Exhaustion Scale (KES)	X		
	Questionnaire	Level of Personality Functioning Scale – Brief Form (LPFS-BF) 2.0	X		
	Questionnaire	Common Mental Disorders Questionnaire (CMSQ), Questions 1-19	X		
	Questionnaire	Level of functioning measured with the Sheehan Disability Scale (SDS)	X		
	Questionnaire	Return to work self-efficacy (RTW-SE)	X		
	Questionnaire	Disability management self-efficacy measured with University of Washington Self-Efficacy Scale (UW-SES)	X		
	Questionnaire	The Quality of Life Scale (QOLS)	X		
	Questionnaire	Health-related quality of life measured with EQ-5D-5L	X		

*RTW* return to work

#### Participant timeline {13}

The participant timeline is shown in Fig. 1.

#### Sample size {14}

Based on a sample size calculation, 900 participants must be included in the trial. On the assumption that even a few weeks’ differences in work per year can have a relevant economic significance, we choose to apply a conservative estimate of a relevant difference between the control and intervention group with a HR of 1.5. The number of days before returning to work is also conservatively estimated at 300 days. With an inclusion period of 1 year and 11 months (700 days), a follow-up time of 365 days, and a 1:1 ratio in the two arms of the trial, a total of 900 participants should be included, to reject the null hypothesis that the time to work of the experimental and control groups is equal with a probability (power) of 94% and a type I error of 0.05. With the inclusion of 900 participants, it is possible with sufficient power (0.94) to examine a HR of 1.25 relative to the two main arms of the trial. A total of 358 participants should be included in each of the subgroups of depression and stress, to reject the null hypothesis that the time to work of the experimental and control groups is equal with a probability (power) of 88% and a type I error of 0.05. The results from the pilot study of IBBIS-II showed that it is unlikely to include enough participants in subgroups of anxiety, personality disorder, and functional disorder to conduct subgroup analyses with enough power.

Power estimation of the secondary outcome, proportion in competitive employment during the 12-month follow-up period, shows that a clinically relevant significant difference of 15% (60% vs. 75%) can be detected with power very close to 1.00, if 450 participants are included in each group. When comparing subgroups, a clinically relevant difference of 15% can be detected with a power of 0.8, if 151 participants are included in each group, meaning that it is likely that there is sufficient power in the depression and stress subgroups to detect relevant differences in employment rates.

#### Recruitment {15}

Case managers from the job centers can refer adult Danish-speaking citizens on sick leave from either work or unemployment to a psychiatric assessment if either the case manager, the citizen, or the individual’s general practitioner (GP) suspects a mental health condition to have caused the sick leave. The referral and assessment are voluntary. The results of the psychiatric assessment will be used in the treatment plan if the participant is allocated to treatment in IBBIS-II. The results of the assessment will be shared with the individual’s GP and the job center. The psychiatric assessment is based on three sources of information about the participant:

1. Personal interview conducted by a care manager/psychologist and supervised by a psychiatrist, guided by the following instruments:
  - Mini-International Neuropsychiatric Interview (MINI), modified to also including stress and burnout [9]
  - Standardized Assessment of Personality – Abbreviated Scale (SAPAS) [11]
  - Level of Personality Functioning Scale – Brief Form (LPFS-BF) [12]
  - Common Mental Disorders Questionnaire (CMSQ) (questions 1–19) [13]
  - Attention-deficit/hyperactivity disorder symptom checklist for adults (Adult ADHD Self Report Scale version 1.1) [14]
  - If dementia is suspected, Mini-Mental State Examination (MMSE)
2. Self-assessed symptoms, e.g., the Four-Dimensional Symptom Questionnaire (4DSQ) [15]
3. GP’s sick leave note

Assessors are IBBIS-II team members who are specially trained to use the abovementioned instruments. Trial eligibility will be evaluated after the psychiatric assessment, and subsequently, assessment data will constitute the baseline data (see Table 1). The assessment process should not take more than 2 weeks but can be prolonged if one or more of the three types of information are missing.

### Assignment of interventions: allocation

#### Sequence generation {16a}

The allocation ratio between the three arms is 1:1. Centralized randomization will take place according to a web-based, computer-generated allocation sequence with varying block sizes kept unknown to the assessors. The consulting firm Deloitte is responsible for the randomization. The randomization will be stratified for labor market status (in job/unemployed), diagnosis (personality disorders/functional disorders/stress/anxiety and depression), and residence (Western Denmark/Eastern Denmark).

#### Concealment mechanism {16b}

To ensure concealment, the randomization schedule is stored away from the research team, and the block sizes are not disclosed.

#### Implementation {16c}

Administrative personnel in the IBBIS-II team perform the online randomization, and the IBBIS-II team leader will assign the participant to interventions and professionals.

### Assignment of interventions: blinding

#### Who will be blinded {17a}

Participants in the trial and the personnel applying the intervention cannot be blinded to the group allocation, whereas those who refer participants, data collectors, and evaluators will be blinded both to the allocation sequence and block size of the randomization, to ensure that they cannot determine which group is the intervention group. During the analysis phase, the groups will be assigned other names, e.g., “X” and “Y,” and the evaluator will only have access to information about the group assignment after the analysis phase has been conducted and the conclusion drawn.

#### Procedure for unblinding if needed {17b}

We do not anticipate any requirement for unblinding because all the included follow-up outcomes are register-based meaning that there are no assessment procedures where unblinding can occur. The computer-generated allocation sequence will be kept unknown to the data analysts until all analyses are performed and conclusions are drawn.

### Data collection and management

#### Plans for assessment and collection of outcomes {18a}

The participants will be followed up at 6 and 12 months using register-based data. The primary, secondary, and explorative outcomes are presented in Table 1. The primary outcome is RTW measured by time to RTW from baseline until 12 months after baseline using register-based employment data (DREAM database). Time to RTW is defined as a minimum of 4 weeks of continuous work without receiving sickness benefits. The secondary outcome is the proportion of unsupported competitive employment at 12 months follow-up using DREAM data. Explorative outcomes include measures of labor market attachment using DREAM data and electronic income data, as well as primary and secondary healthcare using health care data from the National Danish registers (Table 1). The Four-Dimensional Symptom Questionnaire (4DSQ) is a 50-item questionnaire designed to assess self-reported common psychological symptoms in the last week and has a special focus on distinguishing general distress from depression, anxiety, and somatization [15]. The Work and Social Adjustment Scale (WSAS) [16] is a simple, reliable, five-item scale that measures functional impairment related to an identified problem, which is defined in this trial as psychological symptoms. The Karolinska Exhaustion Scale (KES) 26-item version measures the degree of exhaustion disorder and the four interrelated dimensions of exhaustion disorder according to the Swedish National Board of Health and Welfare: lack of recovery, cognitive exhaustion, somatic symptoms, and emotional distress [17]. The Level of Personality Functioning Scale – Brief Form (LPFS-BF) 2.0 measures impairment in personality functioning [12]. The Common Mental Disorders Questionnaire (CMDQ), questions 1–19, is covering symptoms and signs commonly associated with somatoform disorders and worries about health validated as a diagnostic aid in primary care settings [13]. The Sheehan Disability Scale (SDS) measures functional impairment in three inter-related domains: work/school, social life, and family life [18, 19]. Return to work self-efficacy (RTW-SE) is an 11-item measure for self-efficacy beliefs regarding return to work where respondents are asked to respond to statements about their jobs, imagining that they would start working tomorrow in their present emotional state [20]. The 17 items University of Washington Self-Efficacy Scale (UW-SES) measure self-efficacy and is validated across diagnostic conditions [21]. The Flanagan QOLS is a 16-item instrument that measures 5 conceptual domains of quality of life: material and physical well-being; relationships with other people; social, community, and civic activities; personal development and fulfillment; recreation; and independence [22]. The EQ-5D-5L is a measure of health status in five domains: mobility, self-care, usual activities, pain/discomfort, and anxiety/depression and also includes a visual analog scale from 0 (worst imaginable health status) to 100 (best imaginable health status) [23].

#### Plans to promote participant retention and complete follow-up {18b}

All analyses are based on the intention-to-treat principle, and because all outcome measures are exclusively based on register-based data, there will be near to complete follow-up data. Only if a participant wants to discontinue both the intervention and the experiment the data will not be used.

If participants want to discontinue the intervention, there are two options:

1. They discontinue the intervention, but not the experiment, i.e., data can be used in the research project.
2. They discontinue both the intervention and the experiment and data cannot be used and must be deleted.

#### Data management {19}

The data management is handled by the private company Deloitte, Department of Artificial Intelligence & Data. All electronic data (self-assessment, interview, and register data) are stored on secured servers at closed networks, and access to data is logged through a unique login for an assigned list of IBBIS-II personnel. Physical data material (case report forms with selected interview data) is stored in locked spaces in locked facilities. Transfer of electronic data between staff members and other approved data managing institutions is carried out using only tunnel encrypted email or encrypted USB sticks (Table 2).

**Table 2:** Enrollment and data collection

	Baseline	Randomization	6-month follow-up	12-month follow-up
Informed consent	X			
Case report form	X			
Randomization database		X		
Self-assessment data	X			
Register data			X	X

#### Confidentiality {27}

All data will be stored following the European General Data Protection Regulation, as well as national guidelines. Personal information on participants is entered directly into an electronic case report form administered by Deloitte. Only data on participants who have consented to participate in the trial will be stored. At the termination of the trial, data will be transferred to the national archives in accordance with Danish legislation.

#### Plans for collection, laboratory evaluation, and storage of biological specimens for genetic or molecular analysis in this trial/future use {33}

This trial does not involve collecting biological specimens for storage.

## Statistical methods

### Statistical methods for primary and secondary outcomes {20a}

The primary aim is to test whether there is a difference in time to RTW, between the two groups, during the 12-month follow-up period. Since the primary outcome is analyzed exclusively with register data, data is expected to be complete. Kaplan-Meier survival curves will be presented, and the differences between the two intervention groups will be analyzed with a Cox proportional hazards regression. The treatment effect estimates are presented with a hazard ratio (HR) at 95% confidence intervals (CI). Also, Cox regression analysis will be used to analyze the secondary outcome, time from RTW, to any possible new period of sick leave of more than 4 weeks in the 12-month follow-up period.

All continuous outcome measures, including the number of weeks at work and wage income during the follow-up period, will be analyzed by linear regression and presented with mean differences and 95% CI. If data is skewed, a non-parametric test will be used. The dichotomous outcome, employment at one point in the follow-up period, will be analyzed with logistic regression and presented with percentages, odds ratio, and 95% confidence interval. All models will be adjusted for the stratification variables, as well as previous work history and history of social benefits, where a bivariate duration model will be used (number of days in employment or public benefit 5 years before baseline). All analyses will be conducted separately for the two independent randomizations associated with the pilot study and main trial, respectively, as well as collapsed. Subgroup analyses will also be performed (see additional analyses). Only the primary and secondary research questions are considered confirmatory. All other endpoints are considered exploratory, and no adjusting for multiple testing will be introduced.

### Interim analyses {21b}

No interim analyses will be performed in the trial.

### Methods for additional analyses (e.g., subgroup analyses) {20b}

All outcomes are analyzed in subgroup analyses consisting of the following:

- Diagnosis subgroups: anxiety, depression, stress, and functional and personality disorder. Participants from Western and Eastern Denmark
- Self-efficacy: participants with low (≤ 8) or high (> 8) baseline self-efficacy measured by the UW-SES
- People on sick leave from work or unemployment
- Benefits received after sickness benefit, measured at 12 months follow-up and stratified by unemployment benefits, subsidized “flexjobs,” work subsidy in private and public companies, cash benefits, and SU (the Danish students’ Grants and Loans Scheme)

Moreover, the participants from the pilot phase will be included in the additional sensitivity analysis on the included outcomes.

An elaborate data analysis plan will be prepared before the analytic phase and will be uploaded to ClinicalTrials.gov.

### Methods in analysis to handle protocol non-adherence and any statistical methods to handle missing data {20c}

The data analyses are based on the intention-to-treat principle. Data from all participants will be included in the analyses, whether they complete the intervention. Incomplete baseline data will be handled using multiple imputation assuming that data is missing at random.

### Plans to give access to the full protocol, participant-level data, and statistical code {31c}

We give access to the full protocol via ClinicalTrials.gov. Access to participant-level data is not applicable due to Danish data protection law.

### Oversight and monitoring

#### Composition of the coordinating center and trial steering committee {5d}

DISCUS has the overall administrative responsibility for the implementation and the operational part of the project. The project is organized with a steering group represented by DISCUS, vocational rehabilitation team members (municipalities), treatment team members (regions), evaluators, and STAR. The steering group meets quarterly to discuss and find solutions to the challenges that may have an impact on whether the project can be carried out as planned and described in the manual and protocol. Deloitte is responsible for data management, randomization, and data collection. The team leaders at the study site are responsible for the day-to-day support for the trial. At CORE, the evaluation is headed by the trial sponsor, who carries the responsibility for the data analysis and evaluation. Metrica are advisors on the design of the evaluation and data analysis.

Deloitte will have authority over data collection. CORE will have authority for the analysis and interpretation of the data, writing of the report and articles, and decision to submit the report and articles for publication. DISCUS, Deloitte, and STAR will not take part in decisions regarding data analysis, nor the interpretation of the publication of results.

#### Composition of the data monitoring committee, its role, and reporting structure {21a}

Data Monitoring Committee was not considered because the trial was considered a low-risk intervention.

#### Adverse event reporting and harms {22}

The services offered are not considered to be associated with any side effects for the participants. It is estimated that participation in the IBBIS-II trial may have a minor strain on the participant by answering questionnaires and participating in interviews. The necessary considerations will be taken in this regard.

#### Frequency and plans for auditing trial conduct {23}

The trial steering group and project management meet every 6 months to review the conduct throughout the trial period.

#### Plans for communicating important protocol amendments to relevant parties (e.g., trial participants, ethical committees) {25}

Important protocol modifications will be notified to the relevant parties, including the steering committee and ClinicalTrials.gov. Any deviations from the protocol will be fully documented using a breach report.

#### Dissemination plans {31a}

The results on time to RTW at 6 and 12 months follow-up, as well as all secondary and exploratory outcomes, will be published in international peer-reviewed scientific journals. Positive as well as negative and neutral results will be published. Authorship is determined by the Vancouver Criteria (http://www.icmje.org). Besides publication in scientific journals, the results will be communicated at relevant scientific meetings and international research conferences. In all dissemination activities, the role of STAR as a donor will be included.

## Discussion

This paper describes the study protocol of a randomized controlled trial comparing (1) IBBIS-II intervention with (2) SAU, i.e., standard mental health care and standard vocational rehabilitation for people on sick leave because of depression, anxiety, personality disorders, or functional disorders. Common mental disorders are frequent causes of sick leave with a great cost for the individual and society. The IBBIS-II trial will test the effect of an integrated health care and vocational rehabilitation intervention to reduce the burden of depression, anxiety, personality disorders, or functional disorders.

This randomized controlled trial is designed with great emphasis on minimizing bias, and reporting is done in accordance with the SPIRIT guidelines [24]. This study has several methodological strengths, including (1) the sample size is large, and hence, we expect high statistical power, which allows for the detection of relevant differences in both primary and secondary outcomes; (2) the randomization is done in accordance with high methodological standards; and (3) the primary outcome is based on register data, and we thus expect complete data on return to work and to avoid the common biases resulting from self-assessed data such as recall bias.

There are nonetheless some limitations. First, participants and professionals are not blinded to the group allocation, and there is, therefore, a risk of both performance bias and subject expectancy bias. These likely biases are difficult to prevent and will be included in the interpretation of the results. Second, several context factors affect the implementation of the intervention, and some variation in the delivered services between the Danish municipalities is expected. We are attempting to minimize the bias from the possibly skewed implementation by stratifying the randomization for municipality. To address the possible differences in the effects between municipalities, we will also conduct fidelity reviews to explicate differences in implementation. Third, standard mental health care and standard vocational rehabilitation for people with stress-related and functional disorders are very scarcely described in Denmark. Thus, a limitation of the study design is the limited knowledge about the quality and quantity of the control intervention. Lastly, the IBBIS professionals are attached to different organizations and are thus subject to different legislative regimes, and journaling must be conducted in different systems. IBBIS team members’ only means of sharing written communication is therefore through emails, which can be seen as a barrier to cross-disciplinary communication and thus a limitation in the implementation of integrated services. Nonetheless, the co-location of IBBIS team members is emphasized in the IBBIS-II model to promote frequent and problem-solving face-to-face communication between professionals and to enhance shared goals, shared knowledge, and mutual respect. If this trial shows that the integrated IBBIS-II mental health care and vocational rehabilitation intervention are superior to SAU, the positive results will support the assumption that integrated care is not only a perceived need from the target group but also an effective way of supporting people in their vocational recovery. This study can contribute new knowledge on integrated vocational and health care interventions in welfare societies with separate health care and occupational sectors, as well as prevention of recurrent sickness absence among people with depression, anxiety, personality, or functional disorders in general. If the IBBIS-II intervention proves to be superior to standard services, the findings can urge policymakers in similar contexts to collaborate on seeking solutions across sectors when the economic benefits from an improved return to work accrue to the social/vocational sector or the employer, whereas the costs of improving access to therapy are placed within the health care sector.

## Trial status

The IBBIS-II trial started on June 9, 2020, and trial recruitment ended on October 1, 2021. A total of 900 participants were included in the trial.

## Data Availability

The final trial data cannot be supplied on request because the data used for the follow-up outcomes are register-based and hosted by Statistic Denmark. The data will only be available to the researchers connected to the project, and it is not possible to download the final trial data set.

## Abbreviations

4DSQ: Four-Dimensional Symptom Questionnaire
CMSQ: Common Mental Disorders Questionnaire
CONSORT: Consolidated Standards of Reporting Trials
CORE: Copenhagen Research Center for Mental Health
DREAM: Danish Register for Evaluation of Marginalization
EQ-5D-5L: European Quality of life – Five Dimensions
IBBIS: Integrated Employment and Treatment Initiative for Sickness Beneficiaries
KES: Karolinska Exhaustion Scale 26
LPFS-BF: Level of Personality Functioning Scale – Brief Form
OECD: Organization for Economic Co-operation and Development
QOLS: Quality of Life Scale
RCT: Randomized controlled trial
RTW-SE: Return to work self-efficacy
SAU: Service as usual
SDS: Sheehan Disability Scale
SPIRIT: Standard Protocol Items: Recommendations for Interventional Trials
STAR: Danish Agency for Labour Market and Recruitment
UW-SES: University of Washington Self-Efficacy Scale
WSAS: Work and Social Adjustment Scale
